# *In vivo* imaging of endothelial cell adhesion molecule expression after radiosurgery in an animal model of arteriovenous malformation

**DOI:** 10.1371/journal.pone.0185393

**Published:** 2017-09-26

**Authors:** Newsha Raoufi-Rad, Lucinda S. McRobb, Vivienne S. Lee, David Bervini, Michael Grace, Jaysree Ukath, Joshua Mchattan, Varun K. A. Sreenivasan, T. T. Hong Duong, Zhenjun Zhao, Marcus A. Stoodley

**Affiliations:** 1 Department of Clinical Medicine, Faculty of Medicine and Health Sciences, Macquarie University, Sydney, New South Wales, Australia; 2 Neurosurgery Department, Inselspital, University of Bern, Bern, Switzerland; 3 Genesis Cancer Care, Macquarie University Hospital, Sydney, New South Wales, Australia; 4 Carestream Molecular Imaging, Sydney, New South Wales, Australia; 5 Department of Physics and Astronomy, Macquarie University, Sydney, New South Wales, Australia; Monash University, AUSTRALIA

## Abstract

Focussed radiosurgery may provide a means of inducing molecular changes on the luminal surface of diseased endothelium to allow targeted delivery of novel therapeutic compounds. We investigated the potential of ionizing radiation to induce surface expression of intercellular adhesion molecule 1 (ICAM-1) and vascular cell adhesion molecule 1 (VCAM-1) on endothelial cells (EC) *in vitro* and *in vivo*, to assess their suitability as vascular targets in irradiated arteriovenous malformations (AVMs). Cultured brain microvascular EC were irradiated by linear accelerator at single doses of 0, 5, 15 or 25 Gy and expression of ICAM-1 and VCAM-1 measured by qRT-PCR, Western, ELISA and immunocytochemistry. *In vivo*, near-infrared (NIR) fluorescence optical imaging using Xenolight 750-conjugated ICAM-1 or VCAM-1 antibodies examined luminal biodistribution over 84 days in a rat AVM model after Gamma Knife surgery at a single 15 Gy dose. ICAM-1 and VCAM-1 were minimally expressed on untreated EC *in vitro*. Doses of 15 and 25 Gy stimulated expression equally; 5 Gy was not different from the unirradiated. *In vivo*, normal vessels did not bind or retain the fluorescent probes, however binding was significant in AVM vessels. No additive increases in probe binding were found in response to radiosurgery at a dose of 15 Gy. In summary, radiation induces adhesion molecule expression *in vitro* but elevated baseline levels in AVM vessels precludes further induction *in vivo*. These molecules may be suitable targets in irradiated vessels without hemodynamic derangement, but not AVMs. These findings demonstrate the importance of using flow-modulated, pre-clinical animal models for validating candidate proteins for vascular targeting in irradiated AVMs.

## Introduction

Brain arteriovenous malformations (AVMs) are a leading cause of stroke in children and young adults, accounting for 4% of hemorrhagic strokes [1]. AVMs are complex vascular abnormalities, forming tortuous, tangled collections of vessels, characterized by abnormal connections between arteries and veins that lack a capillary network [2]. Treatment options for brain AVMs include surgery, embolization and highly focussed radiation (stereotactic radiosurgery). However, over one third of patients with large and deep AVMs cannot be safely and effectively treated with current methods. New treatments are required for these patients with life-threatening AVMs.

A biological technique that can be harnessed to produce thrombosis and occlusion inside AVM blood vessels is highly attractive. A proposed method to achieve this goal is vascular targeting. This approach has been applied in cancer therapy where unique molecular markers expressed on the surface of tumor vessels are targeted by conjugated antibodies and pro-thrombotic factors to induce intravascular thrombosis [3]. Molecular differences that can discriminate diseased and normal endothelial cells (ECs) is the fundamental criterion for successful vascular targeting. Previous studies have shown that AVM ECs are not dramatically different to normal ECs although there are some distinctions. *In vitro* studies suggest a pro-angiogenic phenotype of AVM ECs in culture [4, 5]. Hemodynamic derangements causing turbulent flow within the tangle of AVM vessels are also postulated to modulate expression *in situ* [6]. Targeting moderately expressed proteins in AVMs may not provide sufficient discrimination from normal vessels, further, flow-regulated proteins may also be expressed on AVM feeding arteries. While AVM vessels typically bypass the brain, forming arteriovenous shunts, branches deriving from the main feeding artery can supply blood to the brain, hence their inadvertent occlusion must be avoided.

We previously proposed that stereotactic radiosurgery could be used to prime AVM endothelium, inducing focal molecular changes that could better discriminate the AVM vessels from normal vasculature [7, 8]. We hypothesize that target molecule priming may be possible at lower radiation doses than those currently used clinically for AVM occlusion. Lower doses may allow treatment of large AVMs that are currently untreatable by radiosurgery, because the high doses required to directly induce occlusion lack result in poor radiation drop-off and off-target damage.

Previous studies have shown that radiation can induce endothelial membrane changes such as up-regulation of various cell adhesion molecules, including intercellular adhesion molecule 1 (ICAM-1) and vascular cell adhesion molecule 1 (VCAM-1), both *in vitro* and *in vivo* [9–16]. In addition, both these cell adhesion molecules have been well-explored as vascular targets (reviewed in [17]) in the context of cancer [18]; the endothelial delivery of anti-thrombotic agents [19]; and for targeting endothelial inflammation [20]. These molecules may therefore provide potential candidates for vascular targeting of irradiated AVMs. The aims of this study were firstly to examine the expression level of these molecules in cultured brain ECs over a range of radiation doses (5, 15 and 25 Gy) and to determine the lowest dose able to elicit a significant response. Secondly, we aimed to deliver this dose by Gamma Knife Surgery (GKS) to an animal model of AVM to examine the anatomical localization, level and time course of luminal ICAM-1 and VCAM-1 expression using *in vivo* optical fluorescence imaging.

## Materials and methods

### Animal ethics

All animal procedures were approved by the Macquarie University Animal Care and Ethics Committee (Sydney, Australia) (Approval # 2011/011) and performed in accordance with the Australian Code of Practice for the Care and Use of Animals for Scientific Purposes (8th Edition, 2013).

### Cell culture and irradiation

The mouse brain endothelial cell line, bEnd.3 (ATCC) [21], was cultured in Dulbecco’s modified Eagle’s medium containing L-glutamine (2 mM), sodium pyruvate (110 mg/L), glucose (4500 mg/L), 10% fetal bovine serum (FBS), penicillin (100 units/mL) and streptomycin (0.1 mg/mL). Cells were maintained at 37°C in 5% CO_2_ and passaged at 80–90% confluence. Cells were irradiated at 50–60% confluence in 8-well chamber slides, 6-well plates or 75 cm^2^ flasks with single doses of 5, 15, or 25 Gy, with a 6MV linear accelerator (LINAC) (Elekta Synergy, Crawley, UK) at Macquarie University Hospital (MUH) as previously described [22]. Non-irradiated cells were used as controls.

### Cell viability assay

Cell viability and death were analyzed at designated time points after irradiation using trypan blue staining and an automated cell counter.

### Real-time polymerase chain reaction (qRT—PCR)

Total RNA was extracted by RNeasy Mini-Kit (QIAGEN, Limburg, Netherlands) and reverse transcribed using the AffinityScript QPCR cDNA Synthesis Kit (Agilent Technologies Inc., CA, USA). qRT-PCR was performed using a Rotor-gene 6000 system (Corbett Research, Limburg, Netherlands). Reactions were performed in triplicate with a final reaction volume of 25 μL: 1.0 μL cDNA; 12.5 μL ImmoMix (Bioline, MA, USA); 0.75 μL forward and reverse primers (GeneWorks, MA, USA); 2.5 μL SYBR Green (Life Technologies, CA, USA); 8.25 μL nuclease-free PCR-grade water. Thermal cycling conditions consisted of denaturation at 95°C for 10 min, followed by 40 cycles of: 95°C for 20 s; 58°C for 20 s; 72°C for 20 s; and one step at 72°C for 7 min. Data were analyzed using the comparative Ct (threshold cycle) method and normalized to the housekeeper hypoxanthine guanine phosphoribosyltransferase (HRPT) [23]. Primer sequences were: ICAM-1 (forward: GCCTCCGGACTTTCGATCTT; reverse: GTCAGGGGTGTCGAGCTTTG); VCAM-1 (forward: GGGAAGCTGGAACGAAGTATCC; reverse: TCTGGAGCCAAA CACTTGACTGT); HPRT (forward: GCTTTCCCTGGTTAAGCAGTACA; reverse: CAAACTTGTCTGGAATTTCAAATC) [24].

### Western blotting

Whole cell protein lysates were prepared using radioimmunoprecipitation (RIPA) assay buffer (150 mM NaCl, 50 mM Tris-HCl, pH 7.5, 0.5% deoxycholate, 0.1% SDS, 1% NP-40 substitute, 5 mM EDTA, pH 8.0, protease inhibitor cocktail (GE Healthcare, NJ, USA)). Protein extracts (15 μg) were run on 10% polyacrylamide gels before transfer and detection with primary antibodies: ICAM-1 (sc-1511R, Santa Cruz, CA, USA); VCAM-1 (ab134047, Abcam, Cambridge, UK) and GAPDH (ab9485, Abcam) and goat anti-rabbit horseradish peroxidase (HRP)-conjugated secondary antibody (ab6721, Abcam). Bands were visualized using enhanced chemiluminescence. GAPDH was used as a loading control. Band intensity was quantified using Image J (http://imagej.nih.gov/ij/download.html, 1997–2014) and normalized to non-irradiated controls.

### Enzyme-linked immunosorbent assay (ELISA)

Cell ELISA was carried out to determine the level of surface expression of endothelial cell adhesion molecules after irradiation. ELISA kits for ICAM-1 (BEK-2024-1P) and VCAM-1 (BEK-2107-1P; Biosensis, SA, Australia) were used according to the manufacturer’s instructions. To account for differences in cell confluence after radiation, the HRP signal was normalized to cell number by measuring absorbance at 595 nm after Janus Green staining (Sigma-Aldrich, MO, USA).

### Immunocytochemistry

Cells were cultured in 8-well chamber slides and irradiated by LINAC at 5, 15 and 25 Gy doses. Cells were fixed 24, 72 or 120 h after radiation with 4% paraformaldehyde prior to blocking and incubation with anti-ICAM-1 antibody (ab119871, Abcam) or IgG2b isotype (ab18541, Abcam) that was fluorescently tagged using Mix-n-Stain^TM^CF^TM^555 antibody labelling kit (Sigma-Aldrich), or anti-VCAM-1 antibody (ab61993, Abcam) or IgG1 isotype (ab18407, Abcam) labelled using Mix-n-Stain^TM^CF^TM^640 antibody labelling kit (Sigma-Aldrich). Nuclei were stained using 4',6-diamidino-2-phenylindole (DAPI) (1 μg/mL, Life Technologies). Images were captured with fixed parameters using a confocal microscope (SP5, Leica Microsystems, Wetzlar, Germany) and analyzed with ImageJ.

### Rat AVM model and Gamma Knife surgery

An arteriovenous fistula (AVF) was created surgically in isoflurane-anesthetized 6-week old, male Sprague-Dawley rats (n = 48) by anastomosing the caudal end of the left external jugular vein to the left common carotid artery as described in detail previously [25, 26]. The AVF was considered ready for Gamma Knife surgery (GKS) six weeks following creation. Anesthetized rats (n = 24) were treated using a single-fraction stereotactic radiosurgical dose administered using a Leksell Gamma Knife Perfexion (Elekta Instruments, Stockholm, Sweden) at Macquarie University Hospital (Sydney, Australia). GKS planning by axial full-body CT scanning with 3D reconstruction and subsequent treatment was performed as described [25], with the exception that a single marginal 15 Gy dose of radiation was delivered.

### Near-infrared (NIR) dye preparation and NIR fluorescence optical *in vivo* imaging

*In vivo* fluorescence optical imaging was performed to localize and quantify luminal expression of ICAM-1 and VCAM-1 in the AVM model. The antibody-dye probe was prepared by conjugating the NIR dye, Xenolight CF750 (Caliper Life Sciences Inc., MA, USA, PK1125674), to antibodies targeting ICAM-1 (#554967, BD Biosciences, CA, USA) or VCAM-1 (#559165, BD Biosciences), or an IgG1 isotype control antibody (#553447, BD Biosciences), and the degree of labelling routinely established at 2–2.2 dye molecules per protein according to the manufacturer’s instructions (Caliper Life Sciences, Inc.). For imaging, each anesthetized rat was placed prone in a Kodak In Vivo Multispectral Imaging System FX cabinet (Carestream, NY, USA). The Xenolight 750-conjugated probes were injected via the tail vein, followed by a NIR fluorescence image (excitation filter, 730 nm; emission filter, 790 nm; exposure time, 60 s; bin = 2 × 2; f-stop = 2.80; field of view = 120 mm). An X-ray image (exposure time, 60 s; f-stop = 2.5; field of view = 120 mm) was taken immediately after to allow anatomical localization of the fluorescence signal. Prior to probe injection, an image in the Xenolight 750 NIR fluorescence channel was taken to obtain a background reading.

Dosing was optimized according to signal intensity and dye clearance from the body. The optimal time for imaging for both Xenolight 750 probes was 12 h post-injection, using a 25 μg/kg concentration of dye conjugate (not shown). A Xenolight 750-conjugated IgG1 mouse isotype control antibody (25 μg/kg) was used as a negative control to account for non-specific binding (n = 6). A total of 28 animals with AVM creation were distributed for Xenolight 750-ICAM-1 or Xenolight 750-VCAM-1 probe testing after sham or GKS. On days 1, 7, 21, 42, 63 and 84 post-irradiation or sham, animals were injected via the tail vein as described. The same animals were recovered after imaging and used across all time points.

### Analysis of *in vivo* fluorescence imaging

Image analysis was performed with ImageJ. Original images were exported as 16-bit files, opened in sequence, converted to stack and the rolling ball algorithm used to subtract background. The images were artificially coloured as “Fire” and stacked images converted to montage with a scale showing high (yellow) and low (purple) fluorescent signal intensity. A region of interest (ROI) was depicted around the AVM area (the “target” area) and an ROI of equal size was depicted on the lateral sides of each rat in the axillary region (“non-target” area). The ROI in pre-injection images was used for background calculation. Mean fluorescence intensity (MFI) for each image was calculated as follows: (target ROI–background ROI)/(non-target ROI–background ROI). Optimization experiments express the data as MFI and are given in arbitrary units. To account for inter-subject variation, MFI data were normalized to matched animal data at day 1 to reduce inter-animal variation in the time course analysis.

### *Ex vivo* fluorescence imaging

Immediately after *in vivo* imaging at day 84, rats were perfused with saline and selected tissues excised. *Ex vivo* NIR fluorescence imaging was performed under the same parameters using the In Vivo Multispectral Imaging System. Selected AVM tissues were the left common carotid artery (CCA), the AVM nidus and the left external jugular vein (EJV); the right CCA and right EJV (contralateral vessels) were used as control tissues.

### Statistical analysis

All data are presented as mean ± standard error of the mean (SEM). Data were plotted and analyzed using Prism v 6.0 (GraphPad). For *in vivo* imaging, Kruskal-Wallis with Dunn’s post-hoc analysis was performed for non-parametric data. Parametric data were analyzed by repeated measures two-way analysis of variance (ANOVA) to consider the effects of treatment and time. For immunocytochemistry, ELISA and qPCR-RT, data were analyzed by two-way ANOVA with Bonferroni corrections. Western data were analyzed by one-way ANOVA with Bonferroni corrections. P values less than or equal to 0.05 were considered significant. *P<0.05, **P<0.01, ***P<0.001, ****P<0.0001.

## Results

### Effect of irradiation on cell viability

**Fig 1:**
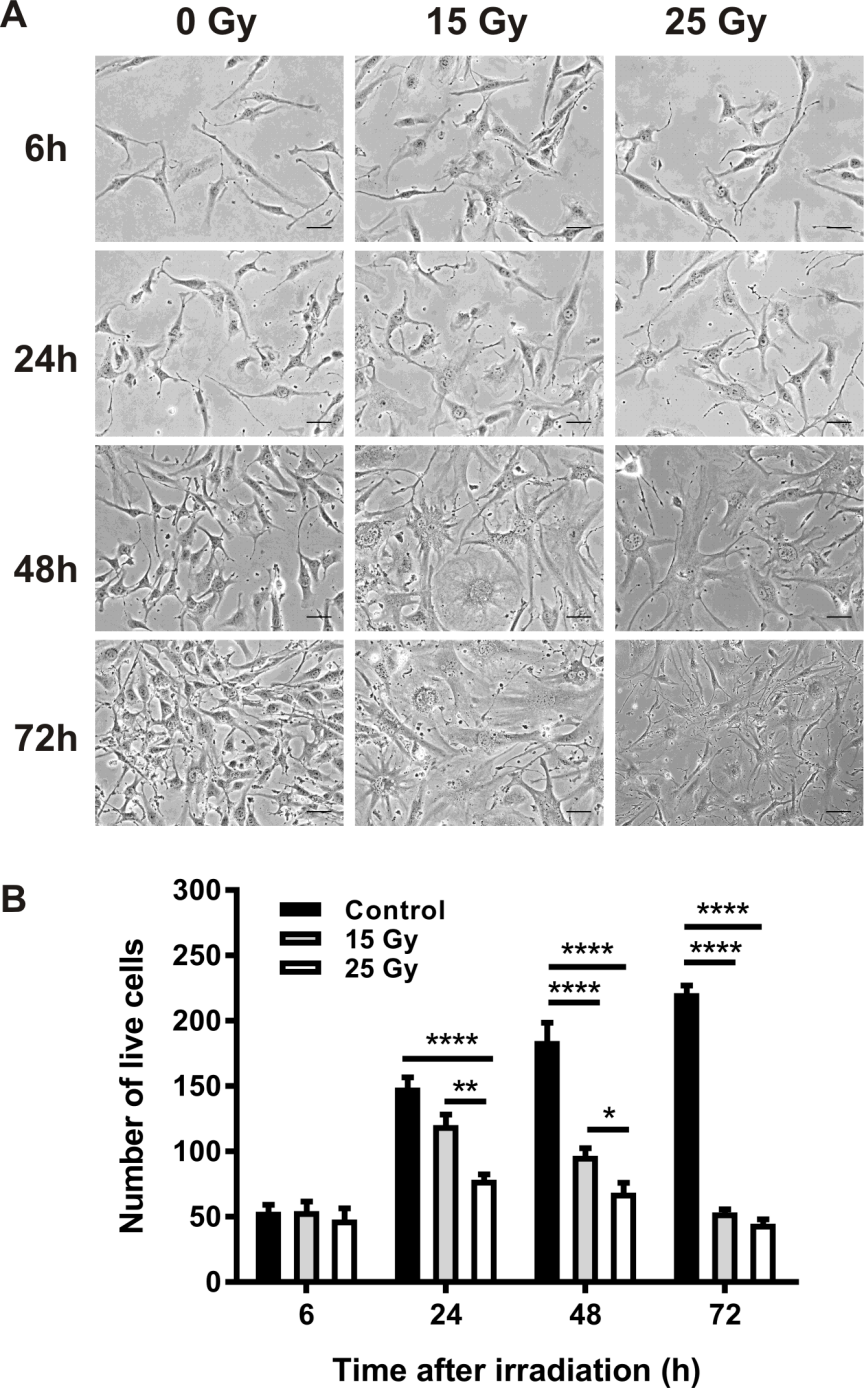
Effect of radiation on bEnd.3 cell morphology and viability. (A) Representative images of bEnd.3 cells after radiation at doses of 15 and 25 Gy. Scale bar = 20 μm. All images at 200× magnification. (B) Cell viability was determined by trypan blue assay. Values are mean ± SEM, n = 3 for each group.

Cell viability was determined at 6, 24, 48 and 72 h after irradiation by automated trypan blue assay. In non-irradiated cells, the number of live cells continued to increase over 72 h (Fig 1A and 1B). In contrast, radiation doses of 15 Gy and 25 Gy significantly reduced the number of viable cells and induced substantial morphological changes in cells remaining adherent after 48 h. Morphological changes were consistent with the development of stress-induced senescence, characterized by cellular hypertrophy and enlarged, often multinucleated or multilobed, nuclei, consistent with previous studies [27, 28].

### Radiation increases ICAM-1 and VCAM-1 gene and protein expression

**Fig 2:**
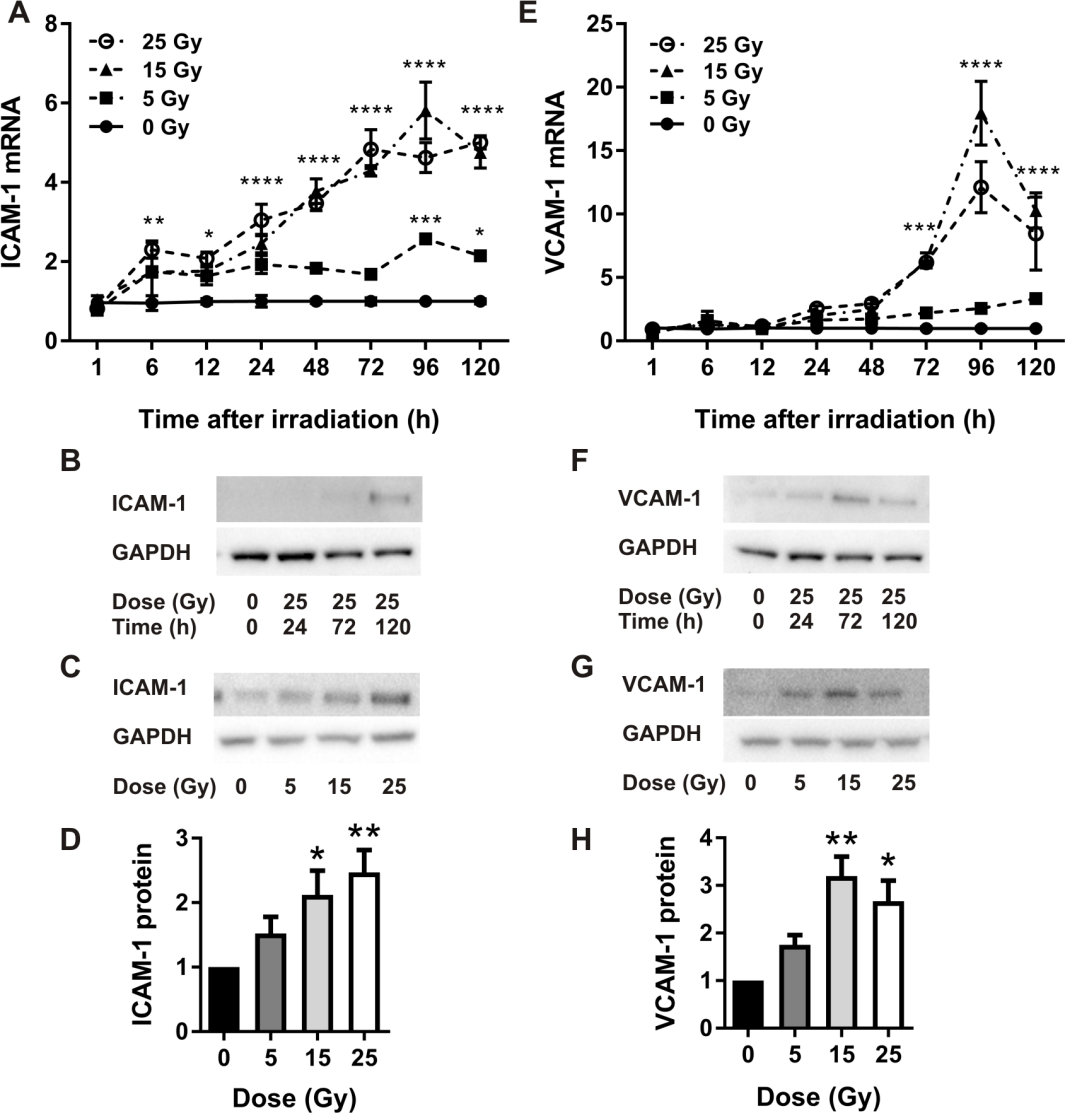
Quantitative real-time PCR and western analysis of ICAM-1 and VCAM-1 expression in irradiated bEnd.3 cells. qRT-PCR analysis of ICAM-1 (A) and VCAM-1 (E) gene expression, n = 4 independent experiments. Representative western blots (time course) of ICAM-1 (B) and VCAM-1 (F) protein at 25 Gy. Representative western blots (dose response) of ICAM-1 expression (120 h) (C) and VCAM-1 expression (72 h) (G) post-irradiation. ICAM-1 (D) and VCAM-1 (H) protein expression quantitated using Image J, n = 4. Values are mean ± SEM. Data were normalized to GAPDH (westerns) or HPRT (qRT-PCR).

Relative gene expression levels of ICAM-1 increased in a linear fashion with time and dose (Fig 2A). There was a significant difference observed between cells irradiated with 5 Gy and cells irradiated with 15 Gy and 25 Gy, but no significant difference between doses of 15 or 25 Gy at any time-point. At the protein level, western blotting demonstrated that ICAM-1 increased linearly with time at a maximum dose of 25 Gy (Fig 2B). At the time of peak expression (120 h), there was a dose-dependent increase in ICAM-1 expression with a significant difference in expression relative to non-irradiated cells at 15 Gy and 25 Gy (Fig 2C and 2D). There was no significant difference between non-irradiated cells and cells exposed to 5 Gy, nor between doses of 15 Gy and 25 Gy, reflective of the gene expression data.

For VCAM-1 gene expression, doses of 15 Gy and 25 Gy significantly increased gene expression relative to non-irradiated cells, peaking at 96 h (Fig 2E). There was no significant induction at a dose of 5 Gy. Expression was marginally higher at 15 Gy relative to 25 Gy but not statistically significant. At the protein level, western blotting analysis demonstrated that VCAM-1 peaked 72 h after irradiation at the maximal dose of 25 Gy (Fig 2F). At the 72 h peak, there was a significant difference between non-irradiated and 15 Gy and 25 Gy treatment groups (Fig 2G and 2H). As noted for gene expression, VCAM-1 protein levels appeared higher in response to 15 Gy compared to 25 Gy, however this did not reach statistical significance.

### Radiation increases cell surface expression of ICAM-1 and VCAM-1

**Fig 3:**
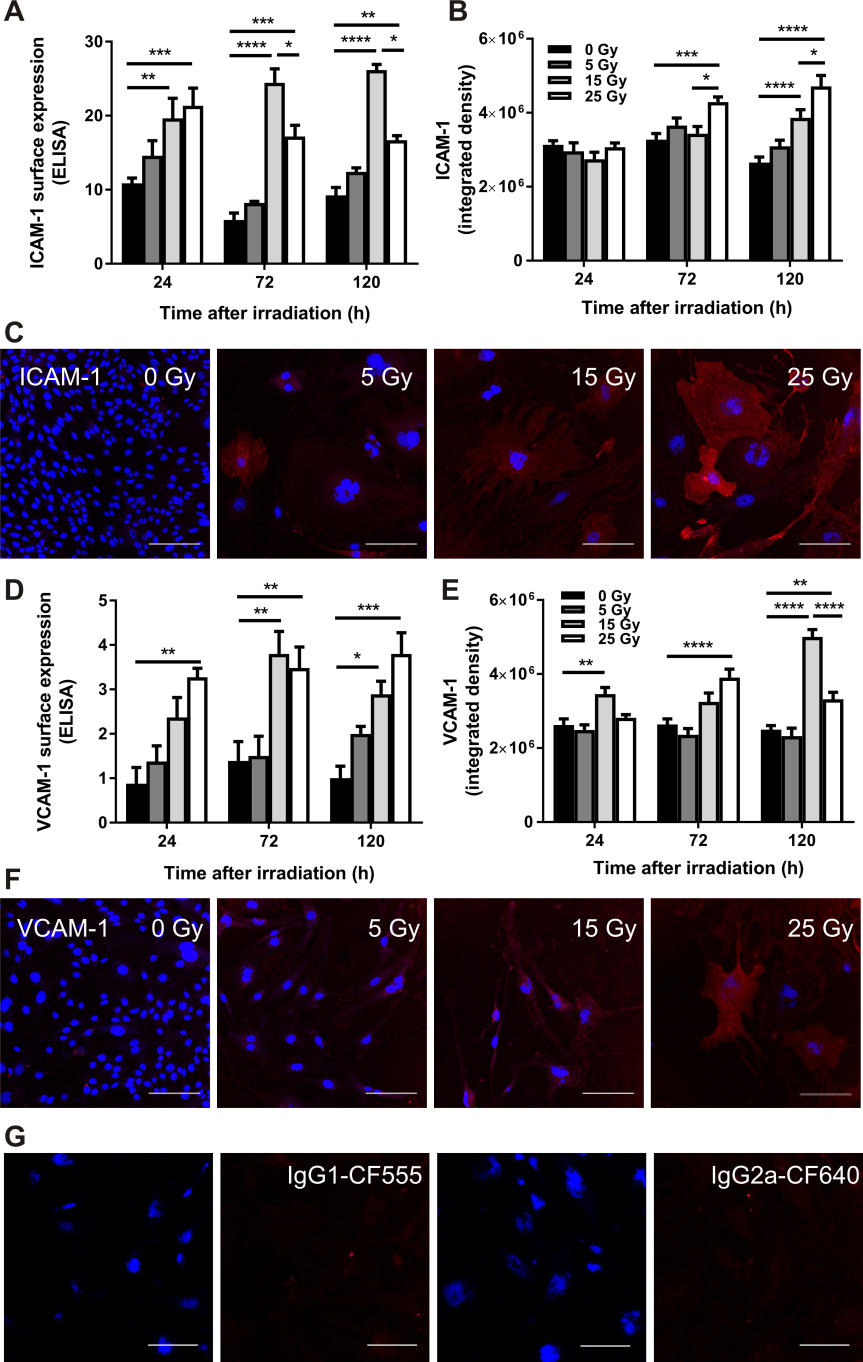
ELISA and immunocytochemical analysis of ICAM-1 and VCAM-1 expression in irradiated bEnd.3 cells. ELISA analysis of surface ICAM-1 (A) and VCAM-1 (D) expression in irradiated bEnd.3 cells normalized to Janus green absorbance to account for changes in cell number. Immunocytochemistry was performed on bEnd.3 cells using CF555-conjugated ICAM-1 or CF640-conjugated VCAM-1 antibodies (red). Staining was quantitated as integrated density using Image J (arbitrary units) for ICAM-1 (B) and VCAM-1 (E). Representative images are shown at 120 h (ICAM-1) (C) or 72 h (VCAM-1) (F) post-radiation at doses of 0–25 Gy. Isotype controls for ICAM-1 (IgG1-CF555) and VCAM-1 (IgG2b-CF640) showed no staining (representative images shown at 25 Gy, 72h). Cells were counterstained with DAPI to visualize nuclei (blue). All images were acquired at a magnification of 200× (scale bar = 100 μm). Values are mean ± SEM, n = 3 for each group.

To ensure the gene and protein expression data for ICAM-1 and VCAM-1 after irradiation correlated with surface translocation, an important factor for *in vivo* vascular targeting, whole-cell ELISA assays and immunocytochemistry were used to examine surface expression. Consistent with the qRT-PCR and western blot results, there was a marked increase in cell surface expression of ICAM-1 in adherent bEnd.3 cells post-irradiation as detected by ICAM-1-specific ELISA (Fig 3A). Expression increased dramatically in irradiated cells at doses of 15 Gy and 25 Gy however there was no significant difference at a dose of 5 Gy relative to non-irradiated cells. A dose of 15 Gy showed higher surface expression of ICAM-1 compared to 25 Gy. Immunocytochemical analysis showed that ICAM-1 was low at early time-points and in the absence of radiation treatment but increased over time and with radiation dose (Fig 3B and 3C), in association with the senescence-like morphological enlargement of the remaining adherent cells [27, 28]. Cells irradiated with 15 and 25 Gy showed a significant elevation compared to non-irradiated cells. There was no significant difference between a dose of 5 Gy and non-irradiated control cells.

ELISA quantitation of VCAM-1 cell surface expression demonstrated that VCAM-1 was significantly elevated in response to irradiation with 15 Gy and 25 Gy doses (Fig 3D). No significant increase was evident in cells dosed at 5 Gy, nor was there a significant difference between 15 Gy and 25 Gy. Immunocytochemical analysis showed that VCAM-1 was poorly expressed at early time-points and in the absence of radiation but increased with dose and time (Fig 3E and 3F). Cells irradiated at 5 Gy showed no significant difference to non-irradiated cells. At the 120 h time-point, VCAM-1 expression was higher in response to 15 Gy than 25 Gy, consistent with trends in gene and protein analysis. Elevated VCAM-1 expression was also associated with morphological changes, including flattening and hypertrophy, characteristic of radiation-induced cellular senescence [27, 28].

### Xenolight 750-ICAM-1 and VCAM-1 probes bind specifically within AVM vessels

**Fig 4:**
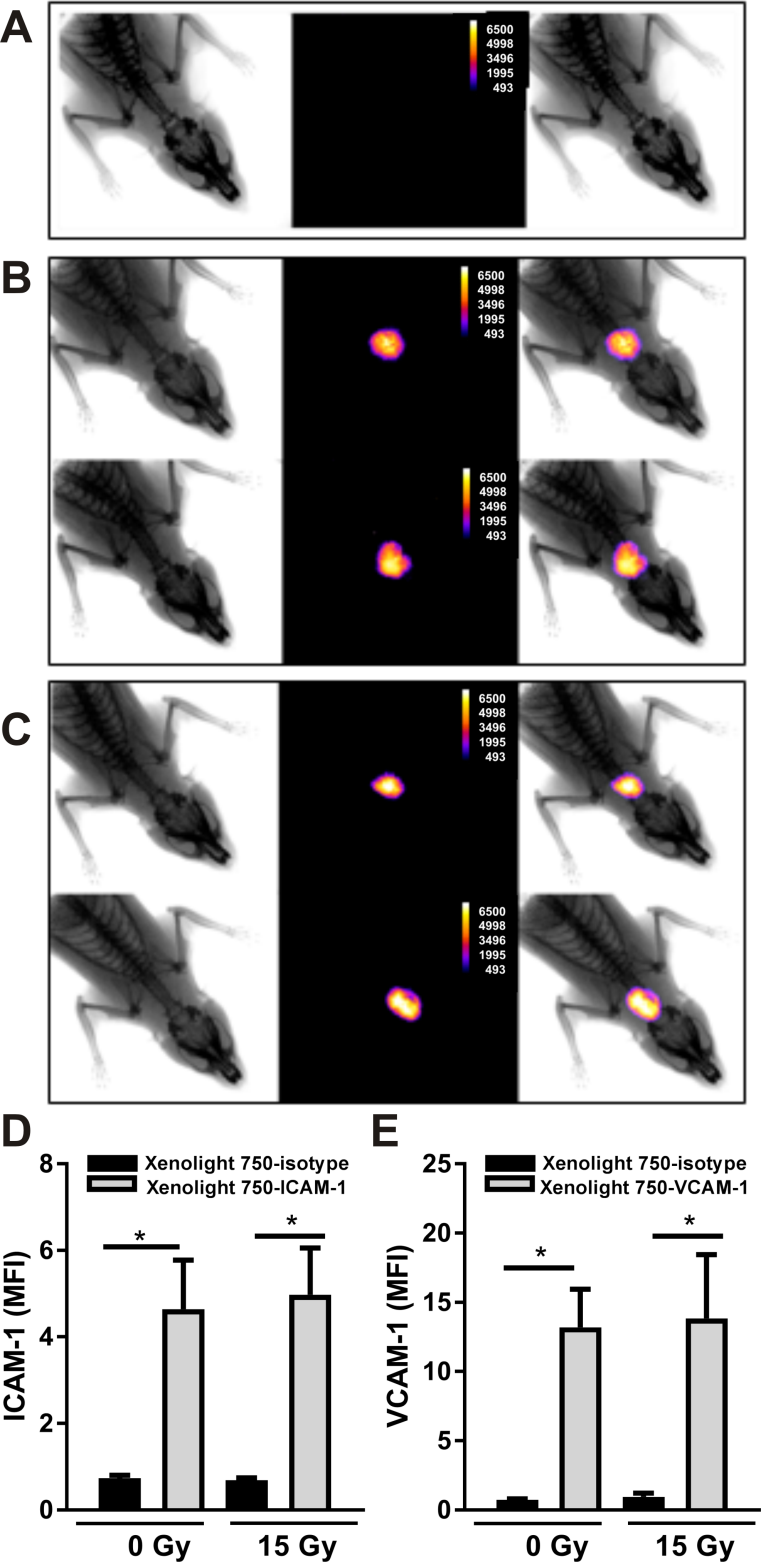
*In vivo* near-infrared fluorescence imaging of Xenolight 750 probes in the rat AVM model. Rats with an AVM creation were sham treated or irradiated with a 15 Gy marginal dose to the AVM region by Gamma Knife and imaging performed 12 h after conjugate dye injection (25 μg/kg). Representative montages of x-ray (left), fluorescent (centre) and merged (right) images after injection of Xenolight 750 probes: (A) Xenolight-750 isotype control in irradiated animal; (B) Xenolight 750-ICAM-1 probe and; (C) Xenolight 750-VCAM-1 probe, at day 21 after sham (top panels) or radiation (bottom panels). Image J quantitation of fluorescence at day 21 post-irradiation or sham with Xenolight 750-ICAM-1 (D) or Xenolight 750-VCAM-1 (E) probes and Xenolight-750 isotype control probe.

Preliminary *in vivo* imaging experiments optimized intensity and clearance with administration of Xenolight 750-ICAM-1 or VCAM-1 probes at a concentration of 25 μg/kg, with images taken 12 h post-injection (not shown). Non-specific binding was examined using the non-targeting Xenolight 750-isotype control injected at day 21 post-irradiation or sham (n = 6, each cohort; 25 μg/kg). No fluorescent signal was detected in response to this non-targeting probe in any animal either sham-irradiated or irradiated (representative image of irradiated animal shown in Fig 4A). In contrast, the Xenolight 750-ICAM-1 probe produced a fluorescent signal localized specifically to the AVM area both in non-irradiated (Fig 4B top panel) and irradiated animals (Fig 4B lower panel) injected at day 21 post-irradiation. Similarly, for the Xenolight 750-VCAM-1 probe injected in non-irradiated (Fig 4C top panel) and irradiated (Fig 4C lower panel) rats at day 21 post-irradiation, fluorescent conjugate accumulated only within the AVM area as found for ICAM-1. Quantification of *in vivo* fluorescence showed a significant 4-fold difference between non-targeting and Xenolight 750-ICAM-1 probes (Fig 4D) and a 13-fold increase between non-targeting and Xenolight 750-VCAM-1 probes (Fig 4E) for both non-irradiated and irradiated rats at day 21 (P<0.05). There was no significant difference between non-irradiated and irradiated animals for either molecule at this time point.

### Expression of ICAM-1 and VCAM-1 is not further modulated by irradiation in the rat AVM

**Fig 5:**
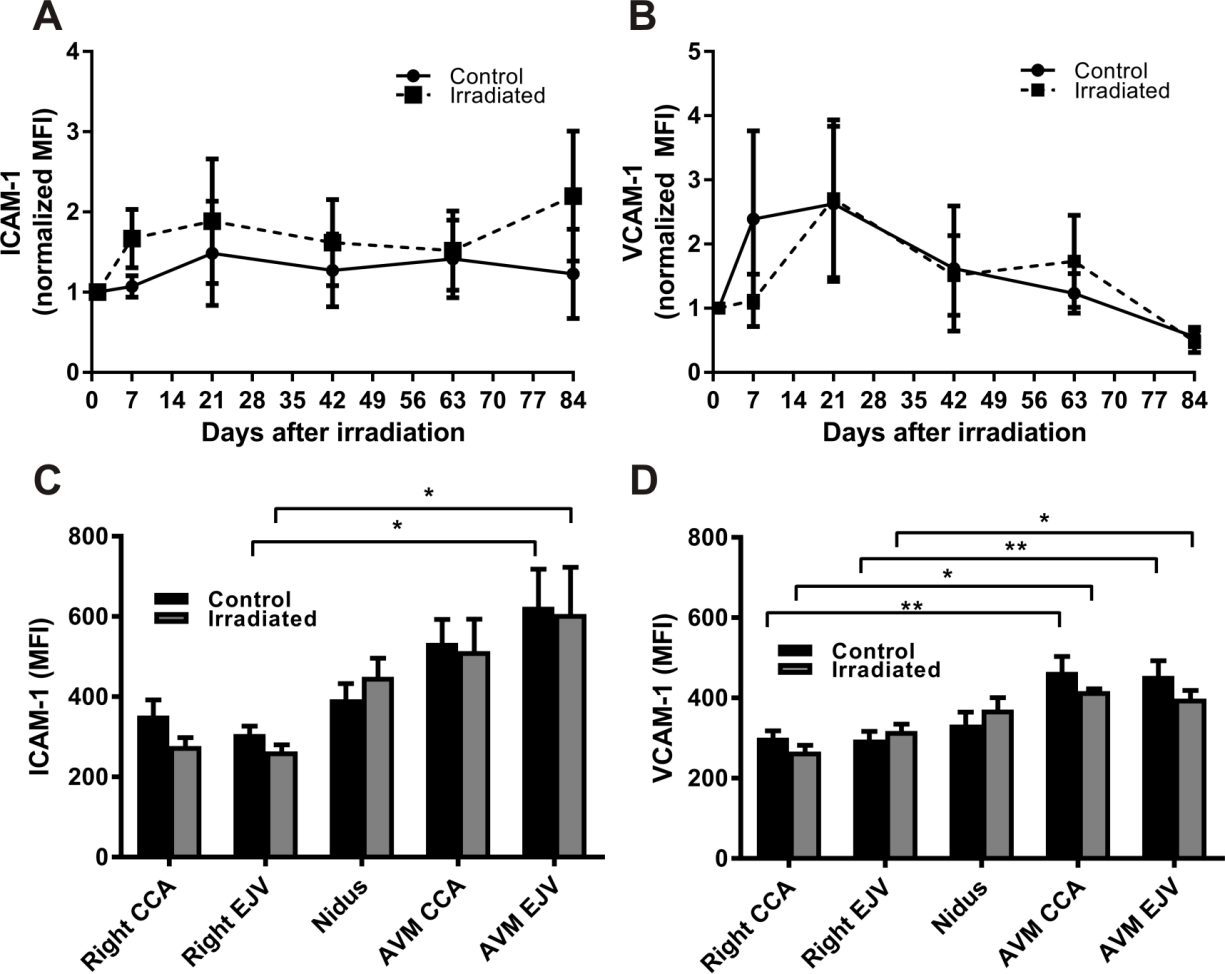
Time course of ICAM-1 and VCAM-1 expression in the rat AVM model. Xenolight-750 probe binding was examined over a period of 84 days for ICAM-1 (A) and VCAM-1 (B) in control and irradiated (15 Gy) animals (n = 7 per group). Raw MFI (mean fluorescence intensity) was normalized to day 1 in matched animals to reduce inter-animal variation. No significant differences were detected at any time between irradiated and control AVMs. *Ex vivo* image analysis of Xenolight 750-ICAM-1 (C) and Xenolight 750-VCAM-1 (D) probe binding in excised tissue: common carotid artery (CCA), external jugular vein (EJV) (n = 7).

*In vivo* imaging was performed over a period of 84 days to study the temporal expression of cell surface ICAM-1 and VCAM-1 post-irradiation with the Xenolight 750 probes. For ICAM-1, basal expression in the non-irradiated AVMs remained high across all time points. Raw data were normalized to MFI values day 1 post-irradiation given there was no significant difference in mean MFI between irradiated and control groups at this time point (mean MFI 4.8 ± 1.2 in sham vs 4.5 ± 0.9 in irradiated animals). When the data were normalized to reduce inter-animal variability and examine expression over time, there was no significant change over the 84 days in sham animals (Fig 5A). After irradiation, a 1.6-fold increase was evident by day 7 which remained stable to day 84, however there was no statistical difference at any time point. Repeated measures analysis did not reveal any significant effect of time or treatment on ICAM-1 expression. *Ex vivo* imaging of excised vessels after probe injection at day 84 showed elevation of Xenolight 750-ICAM-1 binding in the AVM vessels (nidus, CCA and EJV) relative to their respective contralateral controls with statistical significance at the EJV (Fig 5C). There was no significant difference between non-irradiated and irradiated vessels at each site.

For VCAM-1, basal expression in non-irradiated animals was high relative to normal vessels in the AVM region across all time points. Raw data were normalized to MFI values day 1 post-irradiation given there was no significant difference in mean MFI between irradiated and control groups at this time point (mean MFI 5.9 ± 1.0 in sham vs 7.9 ± 3.2 in irradiated animals). In both sham and irradiated animals, normalized mean MFI increased up to day 21 then slowly decreased over the 84 day period (Fig 5B). There was no significant difference between non-irradiated and irradiated animals at any time point, similar to ICAM-1. Repeated measures analysis did not reveal any significant effect of time or treatment on VCAM-1 expression. *Ex vivo* imaging confirmed elevated Xenolight 750-VCAM-1 binding in AVM vessels relative to the respective contralateral vessels, with no significant difference between irradiated and non-irradiated vessels (Fig 5D).

## Discussion

In this study, we examined the potential utility of using cell adhesion molecules ICAM-1 and VCAM-1 as vascular targets in AVMs after radiation priming. *In vitro* we found that radiation doses of 15–25 Gy induce surface expression of ICAM-1 and VCAM-1 in brain microvascular endothelial cells where basal expression is nominal, and that this expression is driven primarily at the level of transcription. Using *in vivo* NIR-fluorescence optical imaging, we found that normal vessels have low endogenous ICAM-1 and VCAM-1 expression, precluding binding of our targeted probes, but revealed that AVMs express high basal levels of these molecules, which was not further enhanced by a single focussed dose of radiation at 15 Gy.

Radiation is well known to induce ICAM-1 and VCAM-1 expression in various cultured cells, including ECs, as also shown in this study. Doses ranging from 2 to 20 Gy have been shown to induce ICAM-1 expression in human umbilical vein ECs (HUVEC) [9, 11, 12]; ICAM-1 expression in human bone marrow ECs [10]; ICAM-1 and VCAM-1 expression in human dermal microvascular ECs (HDMEC) [12, 13]; and VCAM-1 on primary cultures of murine pulmonary endothelial cells [14]. The only previous study performed in a brain endothelial cell line demonstrated a transient ICAM-1 and VCAM-1 up-regulation at a dose of 50 Gy [29]. Our current results show effective doses (15–25 Gy) in brain microvascular EC that fall well within these ranges, but together show that effective dose *in vitro* can vary considerably depending on endothelial cell type, the use of primary versus immortalized cell lines, and on culture conditions.

In vitro, doses of 15 and 25 Gy were equally effective at inducing ICAM-1 and VCAM-1 expression in this study. Given cell viability was higher at 15 Gy relative to 25 Gy, a dose of 15 Gy was chosen for translation to our pre-clinical AVM model, on the assumption that this dose would induce vascular targets while minimizing damage to surrounding cells. Our aim is to identify targetable molecular changes that occur at lower doses than those used currently for clinical induction of AVM occlusion. Single-fraction stereotactic radiosurgery dose regimens demonstrate that for small AVMs, doses within the range of 15–25 Gy are effective with obliteration rates of 72–96% [30–32]. Occlusion rates improve with increasing dose, however a marginal dose less than 15 Gy rarely obliterates AVMs [30]. For larger AVMs, hypofractionated stereotactic radiosurgery dispensing doses in the range of 7 Gy per fraction (4–5 fractions) can achieve occlusion but with higher rates of complication [30, 33]. Applying a lower radiation dose has many clinical advantages. Reducing the dose will increase the size of AVM lesions that can be treated, providing treatment options for patients with large AVMs currently classed as untreatable. Lower doses reduce the likelihood of radiation damage to normal cells surrounding the AVM tissue. Use of irradiation as a vascular primer in non-AVM contexts would also benefit from a minimal dose of radiation for this same reason.

The absence of effect of radiation on ICAM-1 and VCAM-1 expression *in vivo* may be a result of the non-irradiated AVM vessels already expressing a high level of ICAM-1 and VCAM-1 on their endothelial surface. We previously reported on the presence of basal endothelial VCAM-1 expression in this model using immunohistochemical studies [34], where we observed an increase in response to a 25 Gy dose of radiation at day 21. Potentially, the use of 15 Gy rather than 25 Gy in this study reduced the significance of the effect *in vivo*, even though this dose significantly increased expression of ICAM-1 and VCAM-1 *in vitro*. Although radiation may not induce additional expression of ICAM-1 and VCAM-1 over the high basal levels within the context of AVM treatment, the lack of binding in the normal vasculature together with findings from other *in vivo* studies that show significant endothelial induction in non-AVM contexts [14–16] suggests these molecules may be targetable in other pathological contexts, where hemodynamic changes are not inherent.

The presence of high basal ICAM-1 and VCAM-1 and lack of further induction with radiosurgery is an important finding and highlights the importance of validating potential candidates within a suitable pre-clinical model. The created AVM animal model has similarities in hemodynamic, histological, molecular and ultrastructural characteristics to human AVMs [25, 26]. The AVM model consists of a large arterial input, and a nidus with branching vessels that all drain into a single draining vein. Where *in vitro* studies cannot replicate the altered flow conditions and other complexities of the human AVM, the rat AVM model can replicate many of these factors. Previous studies in this AVM model have shown that creation of the rat AVM causes significant hemodynamic changes almost immediately and maintenance of a high flow state [26]. Hemodynamic derangements may be responsible for the increased adhesion molecule expression. *In vitro* studies have revealed that oscillatory shear stress stimulates endothelial ICAM-1 and VCAM-1 expression [6], while laminar shear stress stimulates ICAM-1 but not VCAM-1 expression [35]. The flow-mediated increase in inflammatory molecule expression at the surface of AVM vessels may form part of the propensity of human AVM vessels to rupture or form aneurysms. In the AVM model, we hypothesize that the hemodynamic derangements stimulate significant molecular signalling leading to expression of both ICAM-1 and VCAM-1, precluding further induction in response to the radiation dose delivered in this study.

Given the flow-modulated ICAM-1 and VCAM-1 expression found in the rat AVM model, a question remains as to whether this level of expression occurs in hemodynamically deranged human AVMs. We previously demonstrated faint to moderate expression of ICAM-1 and VCAM-1 in human AVMs using immunohistochemistry [36]. Elevated expression of ICAM-1 and VCAM-1 in AVM tissue could be a result of increased levels of shear stress [35], or high levels of vascular endothelial growth factor or cytokines [37]. The expression of these adhesion molecules in human AVMs is consistent with the elevated basal expression in the rat AVM and further validates the model. It is difficult to determine, however, if the level expressed in human AVMs would be sufficient to discriminate them from normal vessels, as appeared to be the case for the rat AVMs, even in the absence of radiation. Further, we cannot preclude that this expression does not occur in feeding arteries of AVM vessels, which are also subject to high flow. While AVM vessels typically form an arteriovenous shunt, bypassing the brain, feeding arteries may direct branches into the normal brain, and occlusion of these vessels would be detrimental to brain blood supply. Taking this under consideration, to ensure targeting with high specificity, the main criterion in the context of this study must be to identify those candidates that have a significantly higher level of expression after irradiation than the basal level found on AVM vessels. An ideal candidate protein would lack or have very low basal expression in either the human or rat AVM prior to radiation. Thus we conclude that ICAM-1 and VCAM-1 may not be ideal targets in the context of adjuvant treatments for radiation-primed AVMs.

## Conclusions

In conclusion, this study found that ICAM-1 and VCAM-1 are poorly expressed on unstimulated endothelial cells *in vitro* but can be up-regulated and expressed at high levels on the cell surface in response to radiation treatment. While these molecules may provide legitimate radiation-primed targets in other vascular contexts where basal levels are low, the significantly elevated expression in rat AVMs, delineated in this study using *in vivo* optical imaging, and their potential presence in the feeding arteries of human AVMs, suggests they may be less than ideal in this setting. These findings highlight that the use of appropriate animal models that adequately model the hemodynamic derangements of human AVMs are highly important to validating candidate proteins that can adequately discriminate not only AVM vessels from normal vessels, but AVM vessels from feeder vessels or irradiated tissues.
